# Exposure to a social stressor disrupts the community structure of the colonic mucosa-associated microbiota

**DOI:** 10.1186/1471-2180-14-189

**Published:** 2014-07-15

**Authors:** Jeffrey D Galley, Michael C Nelson, Zhongtang Yu, Scot E Dowd, Jens Walter, Purnima S Kumar, Mark Lyte, Michael T Bailey

**Affiliations:** 1 Biosciences Division, College of Dentistry, The Ohio State University, Columbus, USA; 2 Department of Molecular and Cell Biology, University of Connecticut, Storrs, USA; 3 Department of Animal Sciences, The Ohio State University, Columbus, USA; 4 Molecular Research Mr DNA Laboratory, Shallowater, USA; 5 Departments of Agricultural, Food & Nutritional Science and of Biological Sciences, University of Alberta, Edmonton, Canada; 6 Division of Periodontology, College of Dentistry, The Ohio State University, Columbus, USA; 7 Department of Immunotherapeutics and Biotechnology, School of Pharmacy, Texas Tech University Health Sciences Center, Abilene, USA; 8 Institute for Behavioral Medicine Research, Wexner Medical Center, The Ohio State University, 329A IBMR Building, 460 Medical Center Dr, Columbus, OH 43210, USA; 9 Department of Pediatrics, Wexner Medical Center, The Ohio State University, Columbus, USA

**Keywords:** Psychological stress, Gastrointestinal microbiota, *Lactobacillus*, *Lactobacillus reuteri*, Dysbiosis

## Abstract

**Background:**

The microbiota of the mammalian gastrointestinal (GI) tract consists of diverse populations of commensal bacteria that interact with host physiological function. Dysregulating these populations, through exogenous means such as antibiotics or dietary changes, can have adverse consequences on the health of the host. Studies from laboratories such as ours have demonstrated that exposure to psychological stressors disrupts the population profile of intestinal microbiota. To date, such studies have primarily focused on prolonged stressors (repeated across several days) and have assessed fecal bacterial populations. It is not known whether shorter stressors can also impact the microbiota, and whether colonic mucosa-associated populations can also be affected. The mucosa-associated microbiota exist in close proximity to elements of the host immune system and the two are tightly interrelated. Therefore, alterations in these populations should be emphasized. Additionally, stressors can induce differential responses in anxiety-like behavior and corticosterone outputs in variant strains of mice. Thus, whether stressor exposure can have contrasting effects on the colonic microbiota in inbred C57BL/6 mice and outbred CD-1 mice was also examined.

**Results:**

In the present study, we used high throughput pyrosequencing to assess the effects of a single 2-hour exposure to a social stressor, called social disruption (SDR), on colonic mucosa-associated microbial profiles of C57BL/6 mice. The data indicate that exposure to the stressor significantly changed the community profile and significantly reduced the relative proportions of two genera and one family of highly abundant intestinal bacteria, including the genus *Lactobacillus*. This finding was confirmed using a quantitative real-time polymerase chain reaction (qPCR) technique. The use of qPCR also identified mouse strain-specific differences in bacterial abundances. *L. reuteri*, an immunomodulatory species, was decreased in stressor-exposed CD-1 mice, but not C57BL/6 mice.

**Conclusions:**

These data illustrate that stressor exposure can affect microbial populations, including the lactobacilli, that are closely associated with the colonic mucosa. Because the lactobacilli can have beneficial effects on human health, stressor-induced reductions of their population could have important health implications.

## Background

The human body is colonized by an enormous array of microbes collectively referred to as the microbiota. It is estimated that there are approximately ten times more bacterial cells than there are human cells in the human body with microbiota being found in various receptive niches such as on the skin, in the oral and respiratory tracts, reproductive tract, and most numerously, in the gastrointestinal (GI) tract [1,2]. In the GI tract, the microbiota form a community that fully interacts with one another and with the host. The GI microbiota have multiple beneficial effects on their hosts [3-5] and the structure of the GI community can impact these functions. Of the beneficial interactions between microbiota and host, those that involve the immune system are some of the most intensively studied, and several lines of experiments have demonstrated that the GI microbiota and the host immune system are deeply intertwined in each other’s development [6]. For example, germ-free mice, which have never been colonized by any type of microbe, have diminished immune responses compared to mice colonized with a healthy microbiota [7,8]. Mice deficient in Nod2, a gene that encodes for a receptor that is involved in immune recognition of bacterial muramyl dipeptide, develop a unique microbiota that can lead to colonic inflammation when transmitted to wild-type mice [9,10]. It is now well recognized that the GI microbiota have strong effects on immunoregulation, and immune system activity, in turn, helps to shape the GI microbiota [6-9].

It is estimated that over 500 different species of bacteria can colonize the GI tract [11]. Despite this enormous species-level diversity, only a small number of bacterial phyla are represented. The vast majority of bacteria in the human GI tracts belong to the phyla *Firmicutes* and *Bacteroidetes*, with bacteria in the phyla *Actinobacteria* and *Proteobacteria* also comprising a smaller portion of the overall microbiota as well [12]. The GI microbiota of the murine GI tract is similar to that of humans, with the majority of microbes belonging to the major phyla, *Firmicutes* and *Bacteroidetes*[13-15]. These populations shift slowly over time, but their general stability is important to the health of the host. Abrupt changes to the gut microbiota have been shown to potentially lead to serious negative host health outcomes, including diarrhea, opportunistic infections, and obesity [16-18]. Changes to the microbiota can be caused by factors such as antibiotic use and severe enteric infection. Data from this laboratory, as well as others, indicate that exposure to either physical or psychological stressors can also alter intestinal microbe profiles. Stressor exposure early in life has been demonstrated to alter the types and abundance of bacteria found in the intestines. Separating infant monkeys from their mothers to induce a physiological stress response resulted in a significant reduction in the number of total lactobacilli that could be cultured from the stool [19]. Reductions in lactobacilli are meaningful as certain species, including *L. reuteri*, are involved in immunomodulation [20,21]. In rats, separating the pups from their mothers during the first 14 days of life led to an altered GI microbiome [22]. Stressor exposure during adulthood can also impact the stability of the intestinal microbiota. For example, exposing adult mice to a prolonged restraint stressor was shown to significantly alter microbial profiles in the cecal contents [23].

Similar findings have been associated with a social stressor called social disruption (SDR) [15] that involves repeated social defeat as a result of inter-male aggression over a 2 hr period. When repeated over 6 consecutive nights, this stressor induces a physiological stress response marked by the activation of the hypothalamic pituitary adrenal (HPA) axis and the sympathetic nervous system [24]. The study by Bailey et al. demonstrated that this week-long exposure to the stressor can alter the cecal luminal microbiome in outbred CD-1 mice [15]. However, it is not known whether a single 2 hr exposure to the stressor could induce similar alterations, or whether other mouse strains, such as inbred C57BL/6 mice that are widely used in infectious disease research, are affected by stressor exposure. Knowing whether short-lasting stressors can impact the microbiota has translational importance particularly for the inflammatory bowel diseases (IBD) and irritable bowel syndrome (IBS). Patients with IBD often report stressful periods preceding symptom flares [25]. It is not clear whether acute stress can exacerbate IBD symptomatology, but it is recognized that acute stressor exposure is associated with increased visceral sensitivity in IBS [26]. Because alterations in gut microbiota community structure are evident in IBD and IBS [27,28], and because these alterations are thought to possibly contribute to these diseases [29,30], we determined whether a short-lasting stressor was sufficient to impact gut microbiota community structure.

Previous studies assessing the effects of stress on the microbiota have relied on assessment of intestinal lumen or fecal populations [15,23,31]. Studies in healthy individuals as well as patients with IBS, IBD, or hepatic encephalopathy demonstrate that luminal/fecal microbiota are significantly different than mucosa-associated microbiota [32,33]. Because shifts in microbial populations that are in close proximity to the intestinal epithelium are thought to have the strongest effects on host immunity [34], an additional aspect of this study was to determine whether stressor exposure could impact tissue-associated microbial community profiles.

Stressor-induced changes in lactobacilli have reliably been found in laboratory animals as well as in humans [19,35,36]. However, stressor-induced changes in other taxa have not been widely reported. Thus, 454 pyrosequencing was performed on the colonic tissue of inbred C57BL/6 mice to determine whether a single 2 hr stressor exposure could change colonic tissue-associated microbial community profiles. Quantitative PCR (qPCR) was then used to determine whether stressor-induced alterations in relative abundances evident with the 454 pyrosequencing were also evident as alterations in absolute abundance in both inbred and outbred mice.

## Results

### Social stress affects the community structure of the colonic mucosa-associated microbiota

**Figure 1:**
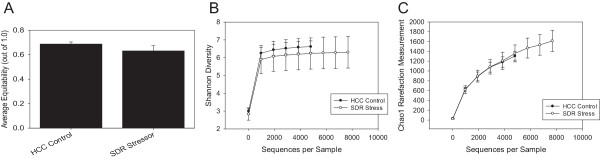
**Exposure to the SDR stressor did not impact alpha-diversity.** Mice were exposed to the SDR stressor, or were left undisturbed as non-stressed Home Cage controls (HCC control). After 454 pyrosequencing, three measures of community alpha-diversity were calculated using QIIME. **A**. Equitability Index, **B**. Shannon Diversity Index, and **C**. Chao1 Rarefaction Measurement were unaffected by exposure to the SDR stressor. Data are from n = 5 mice per group.

**Figure 2:**
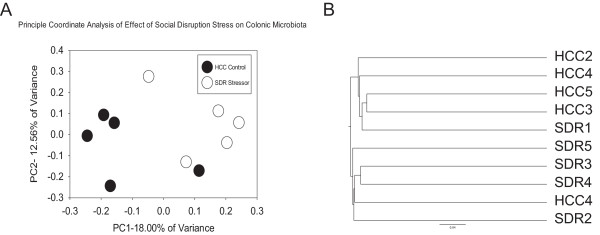
**Exposure to the SDR stressor significantly changes beta-diversity.** After 454 pyrosequencing, UniFrac distances were calculated using QIIME and plotted on a 2-D principal coordinates graph **(A)**. Exposure to the SDR stressor shifted the colonic microbiota, with 4 of 5 SDR stressor samples clustering apart from HCC controls. UniFrac distances were also used for construction of an unweighted pair group method by arithmetic mean (UPGMA) dendrogram, in which 4 of 5 SDR stressor samples clustered together **(B)**. The clustering in Figure 2A and 2B was statistically significant (p < 0.05) after performing the ANOSIM test to measure differences between distance matrices of SDR stressor and HCC control groups. Data are from n = 5 mice per group.

We analyzed the effect of a short term SDR stressor on the colonic mucosa-associated microbiota using 16S rRNA gene pyrosequencing of samples from both SDR C57BL/6 stressor-exposed mice and non-stressed HCC control mice. After normalization of the datasets by rarefaction, we observed no statistical difference in the total number of operational taxonomic units (OTU) between the SDR and control mice (data not shown). Additional analysis of the Shannon, equitability and Chao1 alpha diversity metrics showed no statistical difference between the two groups (Figure 1A-C). Thus, a single two-hour cycle of exposure to the social stressor did not affect the alpha diversity of the mucosa-associated microbiota.While we did not observe any statistically significant differences in the alpha diversity of the microbiota between the SDR and control group, beta diversity analyses did reveal differences in composition. A principal coordinates analysis (PCoA) of the UniFrac distances between samples showed that they clustered into separate groups according to treatment (Figure 2A). An unweighted pair group with arithmetic mean (UPGMA) hierarchical clustering dendrogram confirmed this clustering (Figure 2B). Statistical analysis of the UniFrac distances using the analysis of similarity statistic (ANOSIM) indicated that colonic mucosal microbiota from SDR stressor-exposed mice were significantly different from those of the home cage control (HCC) mice (p < 0.05).

### Populations of the genus *Lactobacillus* and *L. reuteri* are reduced in mice exposed to a social stressor

**Figure 3:**
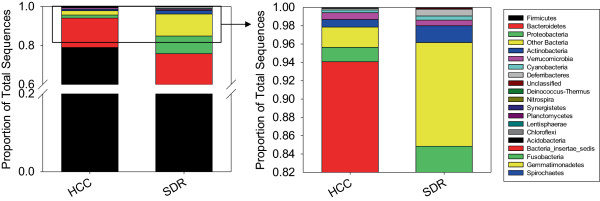
**The relative abundances of mucosal-associated bacterial phyla were unaffected by exposure to the SDR stressor.** Data are the mean+/-S.E. of the relative abundances calculated from 454 pyrosequencing data and are from n = 5 mice per group.

The taxonomic profile of the microbiota at the phylum level showed no significant differences between the two treatment groups (Figure 3). Analysis of relative abundances at lower taxonomic levels showed a reduction in the family *Porphyromonadaceae* in SDR stressor-exposed mice compared to non-stressed HCC mice (p < 0.01) (Table 1). Exposure to the SDR stressor also reduced the relative abundance of bacteria in the family *Lactobacillaceae* (p < .05) (Table 1). This reduction in the *Lactobacillaceae* was reflected by a reduction in the relative abundance of bacteria in the genus *Lactobacillus* (p < .05) (Table 2). In addition to the reduction in the abundance of lactobacilli, it was evident that exposure to the SDR stressor significantly reduced the relative abundance of the genus *Parabacteroides* (p < .01), as well as an unclassified group within the phylum *Firmicutes* (p < .05) and an unclassified group within the class *Bacilli* (p < .05) (Table 2).

**Table 1:** Top 10 most abundant colonic tissue-associated bacterial families

	**HCC**	**SDR**
*Lachnospiraceae*	38.71 ± 5.22	54.10 ± 5.81
*Clostridiales*; Other	9.01 ± 0.56	10.14 ± 0.96
*Bacteroidaceae*	10.77 ± 1.49	6.67 ± 2.11
*Lactobacillaceae*	13.66 ± 4.81	3.13 ± 100*
*Ruminococcaceae*	5.22 ± 0.36	4.35 ± 0.62
*Acetobacteraceae*	0.001 ± 0.004	8.86 ± 8.83
*Clostridiaceae*	6.34 ± 3.62	0.92 ± 0.78
*Porphyromonadaceae*	2.67 ± 0.20	1.59 ± 0.25**
*Bacteria*; Other	2.21 ± 0.25	1.84 ± 0.46
*Peptostreptococcaceae*	1.81 ± 0.35	0.94 ± 0.40

Data are the mean ± standard error. *p < .05 vs. HCC; **p < .01 vs. HCC.

**Table 2:** Top 40 most abundant tissue-associated bacterial genera

	**HCC**	**SDR**
*Lachnospiraceae*; Other	37.61 ± 5.23	53.02 ± 5.74
Clostridiales; Other; Other	9.01 ± 0.56	10.14 ± 0.96
*Bacteroides* spp.	10.77 ± 0.56	6.67 ± 2.11
*Lactobacillus* spp	13.53 ± 4.76	3.11 ± 1.00*
*Rosemonas* spp	0.00 ± 0.00	8.83 ± 8.83
*Ruminococcaceae*; Other	3.81 ± 0.44	2.95 ± 0.45
*Clostridium* spp.	5.86 ± 3.37	0.82 ± 0.70
Unclassified Bacteria	2.21 ± 0.25	1.84 ± 0.46
*Parabacteroides* spp.	2.25 ± 0.12	1.35 ± 0.20**
*Peptostreptococcaceae*; Other	1.80 ± 0.35	0.93 ± 0.40
Unclassified *Firmicutes*	1.25 ± 0.20	0.69 ± 0.13*
*Marvinbryantia* spp.	0.79 ± 0.11	0.81 ± 0.24
*Turicibacter* spp.	0.94 ± 0.30	0.52 ± 0.20
*Oscillibacter* spp.	0.62 ± 0.29	0.65 ± 0.28
*Asaccharobacter* spp.	0.81 ± 0.25	0.39 ± 0.12
*Akkermansia* spp.	0.73 ± 0.32	0.39 ± 0.22
Unclassified *Bacteroidetes*	0.60 ± 0.05	0.33 ± 0.16
*Butyricicoccus* spp.	0.40 ± 0.03	0.52 ± 0.19
Unclassified *Alphaproteobacteria*	0.62 ± 0.40	0.14 ± 0.11
*Erysipelotrichaceae*; Other	0.47 ± 0.14	0.24 ± 0.07
*Butyricimonas* spp.	0.43 ± 0.09	0.22 ± 0.07
*Hyphomonadaceae*; Other	0.01 ± 0.00	0.58 ± 0.55
*Clostridiaceae*; Other	0.48 ± 0.26	0.10 ± 0.08
*Alistepes* spp.	0.38 ± 0.09	0.18 ± 0.05
Unclassified *Cyanobacteria*	0.01 ± 0.00	0.45 ± 0.42
*Chitinophagaceae*; Other	0.39 ± 0.35	0.00 ± 0.00
*Bacillariophyta*; Other	0.06 ± 0.05	0.28 ± 0.17
*Anaerotruncus* spp.	0.15 ± 0.04	0.17 ± 0.02
*Coprobacillus* spp.	0.15 ± 0.05	0.14 ± 0.06
*Mucispirillium* spp.	0.17 ± 0.10	0.11 ± 0.05
*Ruminococcus* spp.	0.22 ± 0.08	0.05 ± 0.03
*Lactobacillales*; Other; Other	0.21 ± 0.07	0.06 ± 0.02
*Blautia* spp.	0.19 ± 0.05	0.07 ± 0.02
Unclassified *Bacilli*	0.18 ± 0.04	0.05 ± 0.02*
*Anaerostipes* spp.	0.16 ± 0.05	0.07 ± 0.01
*Ponticaulis* spp.	0.00 ± 0.00	0.22 ± 0.21**
*Anaerovorax* spp.	0.14 ± 0.05	0.08 ± 0.02
*Holdemania* spp.	0.16 ± 0.06	0.04 ± 0.01
*Bacillus* spp.	0.19 ± 0.19	0.01 ± 0.00
*Roseburi*a spp.	0.09 ± 0.03	0.10 ± 0.04

Data are the mean relative abundance ± standard error. *p < .05 vs. HCC; **p < .01 vs. HCC.

**Figure 4:**
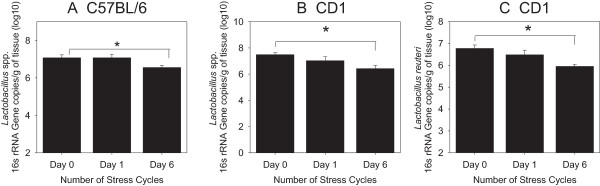
**The absolute abundance of bacteria in the genus *Lactobacillus *is reduced by exposure to the SDR stressor.** Mice were exposed to either 0, 1, or 6 consecutive days of the SDR stressor and qPCR used to quantify *Lactobacillus* group bacteria **(A and B)** or L. reuteri **(C)** in the colon. *indicates p < .05 vs. 0 cycles of SDR (0 cycles is equivalent to HCC controls). Inbred C57Bl/6- n = 16 at Day 0, n = 7 on Day 1, and n = 8 on Day 6. Outbred CD-1- n = 21 at Day 0, n = 15 at Day 1, and n = 6 on Day 6.

Many bacteria in the genus *Lactobacillus* have been shown to have immunomodulatory functions in the colon, and can be used as a probiotic to treat inflammation. Thus, we used qPCR to quantify the effect of the SDR stressor on the abundance of this group of bacteria [20,21]. When the a priori hypothesis that repeated administration of the SDR stressor would reduce the abundance of lactobacilli was tested, it was determined that the absolute abundance of *Lactobacillus* spp. was significantly lower (p < .05) after 6 cycles of the stressor in comparison to baseline levels in C57BL/6 mice (Figure 4A). This reduction in lactobacilli abundance was also observed in outbred CD-1 mice (p < .05), with the largest reduction in mean levels of lactobacilli also occurring after 6 days of the SDR stressor (Figure 4B). In addition, qPCR analyses also revealed that exposure to the SDR stressor significantly reduced the abundance of the immunomodulatory species *L. reuteri* over the course of six cycles of SDR (p < .05). This difference was only evident in outbred CD-1 mice (Figure 4C). L. *reuteri* levels were below the qPCR limit of detection of 4.5 copies/gram of wet tissue (log_10_) in inbred C57BL/6 mice.

### Relative bacterial levels differ by mouse strain

In order to confirm that stressor exposure was reducing the absolute abundance of other bacterial groups that were reduced in relative abundance in the pyrosequencing analysis, qPCR was performed on colonic tissues from both inbred C57BL/6 mice and outbred CD1 mice. Primers targeting the 16 s ribosomal RNA genes of *Parabacteroides distasonis*, a member of the *Parabacteroides* genus, and *Bacteroides-Prevotella-Porphyromonas* were used. Stressor exposure did not significantly affect the absolute abundance of either *P. distasonis* or *Bacteroides-Prevotella-Porphyromonas* genera (Table 3). In addition, levels of *Bacteroides-Prevotella-Porphyromonas* group and *P. distasonis* were similar in the two strains of mice.

**Table 3:** Real-time PCR assessment of bacterial group abundances

		**C57BL6**	**CD-1**
*Parabacteroides distasonis*	Day 0	6.27 (6.02 – 7.42)	6.65 (6.53 – 6.78)
	Day 1	6.94 (6.66 – 7.23)	7.54 (7.30 – 7.77)
	Day 6	6.52 (6.10 – 6.94)	7.00 (6.26 – 7.74)
Group *Porphyromonas-Bacteroides - Prevotella*	Day 0	7.61 (7.39 – 7.85)	7.20 (7.02 – 7.39)
	Day 1	8.10 (7.82 – 8.37)	7.37 (6.93 – 7.82)
	Day 6	7.46(7.17 – 7.75)	6.97 (6.44 – 7.50)

Data are the mean (standard error) of the copy number (log10) of target 16 s rRNA per gram of tissue.

### Colonic inflammatory cytokine mRNA levels are not affected by social stressor exposure

The colonic mRNA levels for IL-1β, TNF-α, and iNOS were unaffected by exposure to the SDR stressor, suggesting that exposing C57BL/6 and CD-1 mice to the SDR stressor did not result in detectable increases in colonic cytokines or inflammatory mediators (Table 4).

**Table 4:** Real-time PCR assessment of colonic inflammation

		**C57BL6**		**CD-1**	
		**DAY 1**	**DAY 6**	**DAY 1**	**DAY 6**
IL1β	HCC	1.00 (0.57 – 1.75)	1.00 (0.55 – 1.82)	1.00 (0.37 – 2.68)	1.00 (0.64 – 1.57)
	SDR	0.95 (0.49 – 1.84)	1.37 (1.00 – 1.37)	3.18 (2.92 – 3.47)	1.11 (0.59 – 2.11)
iNOS	HCC	1.00 (0.56 – 1.78)	1.00 (0.50 – 2.01)	1.00 (0.56 – 1.78)	1.00 (0.69 – 1.46)
	SDR	0.62 (0.46 – 0.83)	2.50 (1.92 – 3.25)	0.32 (0.24 – 0.43)	0.23 (0.14 – 0.37)
TNFα	HCC	1.00 (0.74 – 1.35)	1.00 (0.51 – 1.98)	1.00 (0.76 – 1.32)	1.00 (0.61 – 1.65)
	SDR	0.50 (0.35 – 0.73)	1.03 (0.71 – 1.49)	0.92 (0.69 – 1.22)	0.92 (0.63 – 1.33)

Data are the mean (standard error) of the fold change in cytokine gene expression over non-stressed HCC controls.

## Discussion

Exposure to physical and psychological stressors has been shown to impact the gut microbiota of both laboratory animals and humans [19,22,35]. However, the majority of the previous studies have utilized repeated and prolonged stressors, and have assessed microbial populations in the lumen of the intestines or present in the fecal matter. Whether stressor exposure has different effects on the microbiota of different strains of mice is also poorly understood. Because gut microbes that adhere to the colonic mucosa can have different effects on the host [34], this study assessed whether stressor exposure could alter the community structure of mucosa-associated microbes. This study demonstrated that as little as 2 hrs of stressor exposure is enough to significantly change the structure of the microbiota associated with the colonic mucosa. This effect was not manifest as alterations in alpha diversity, but rather as alterations in beta diversity. The ANOSIM distance matrix analysis and cluster analysis based on unweighted UniFrac demonstrated that microbiota within the samples from stressor-exposed mice were significantly different than those from the non-stressed HCC control mice. In addition to significantly changing beta diversity, exposure to the stressor significantly changed the relative abundance of 2 bacterial genera, namely *Parabacteroides* and *Lactobacillus*, and one bacterial family, *Porphyromonadaceae*.

This study targeted mucosa-associated populations, but true stratification of luminal and mucosal populations does not exist. There is much crossover between microbes often associated with the lumen and those that can adhere to the mucus layer of the GI tract, as the former becomes trapped in the mucus layer and the latter sheds into the lumen. Steps were taken to remove the majority of the fecal matter from tissue samples, wherein lie the majority of the luminal portion of the GI microbiota. This study extends previous studies that indicated that luminal and fecal microbiota can change as a consequence of stressor exposure to now include mucosa-associated populations.

To determine whether the stressor effects only encompassed changes to bacterial relative abundance or whether changes in absolute abundance (as assessed by determining copies of 16 s rRNA gene per gram of sample) may also result from stressor exposure, qPCR was used to further investigate the effects of the stressor on bacterial abundance. This was performed in both an inbred (i.e., C57BL/6) and an outbred (i.e., CD-1) mouse strain, because studies consistently show that microbiota composition is associated with mouse strain [37,38]. In addition, different mouse strains can also have different physiological and behavioral responses to stressor exposure, including changes to anxiety-like behavior, as well as diarrhea output and colonic serotonin concentration [39,40]. Thus, it was important to determine whether stressor effects were conserved across mouse strains. While 2 hrs of stressor exposure was enough to reduce the relative abundance of the genus *Lactobacillus*, a reduction in the absolute abundance was only evident after repeated exposure to the stressor. This indicates that some of the effects that stressor exposure has upon the microbiota are additive. Both pre- and post-stressor lactobacilli levels were similar in outbred CD-1 mice and inbred C57BL/6 mouse strains, demonstrating that the effects of the stressor are consistent across mouse strains. This finding was not surprising given that stressor-induced reduction in lactobacilli levels have been documented in other host species, including human and non-human primates.

The finding that stressor exposure reduced the relative and absolute abundance of lactobacilli is consistent with previous studies demonstrating that stressor exposure can reduce the lactobacilli [19,31,35,36]. To date, however, the *Lactobacillus* species that can be reduced by stressor exposure has not been addressed. Many species of bacteria in the genus *Lactobacillus* are known to have health promoting effects, and studies from this laboratory, as well as others, indicate that administering probiotic *L. reuteri* to mice can reduce colonic inflammatory responses [20,21,41]. Because stressor exposure can exacerbate colonic inflammation [42], we focused on whether indigenous *L. reuteri* was reduced in stressor-exposed animals. As predicted, *L. reuteri* was significantly reduced in colonic mucosa from outbred CD-1 mice exposed to the SDR stressor. Interestingly, however, *L. reuteri* was not consistently detectable in inbred C57BL/6 mice. Thus, it is apparent that *L. reuteri* levels on the mucosa of C57BL/6 mice are lower than levels observed in outbred CD-1 mice and below the detection limit (4.5 log copies/gram of wet tissue).

Although it is difficult to compare deep sequencing results across different experiments that assess different body niches and encompass different stressors, the use of 454 pyrosequencing demonstrated that the effects of stressor exposure are not confined to effects on the lactobacilli. The relative abundance of bacteria in the family *Porphyromonadaceae* was found to be significantly reduced by exposure to the SDR stressor. This finding is consistent with a previous study demonstrating that exposure to a prolonged restraint stressor in oubred CD-1 mice was sufficient to reduce the relative abundance of cecal *Porphyromonadaceae*[23]. However, it was not previously known whether stressor exposure could reduce the absolute abundance of the *Porphyromonadaceae* family. Thus, qPCR was used to determine whether this stressor also affected the absolute abundance of *Porphyromonadaceae* by assessing levels of bacteria in the *Porphyromonas-Bacteroides-Prevotella* group as a surrogate marker. This was performed because of the problems designing qPCR primers specific for the *Porphyromonadaceae* family. The absolute abundance of the *Porphyromonas-Bacteorides-Prevotella* group was similar in CD-1 mice and inbred C57BL/6 mice, and stressor exposure had no effect on the absolute abundance in either mouse strain. Thus, although this study and others [23] have found stressor-induced reductions in the relative abundance of *Porphyromonadaceae*, these results indicate that absolute abundance of the *Porphyromonadaceae* is unaffected by stressor exposure. Stressor exposure also did not affect the absolute abundance of *P. diastonosis* in either strain of mice, even though the relative abundance of *Parabacteroides* was reduced as detected by 454 pyrosequencing.

Colonic inflammation is well known to impact the gut microbiota, and studies have found that intestinal inflammation can also reduce the abundance of the family *Lactobacillaceae*[43,44] and *Porphyromonadaceae*[45]. Given the bidirectional interaction between the microbiota and intestinal inflammation and increased intestinal physiological inflammation upon exposure to stressor [46], it is often suggested that increases in intestinal inflammation during stressful periods could be responsible for changes in gut microbiota. However, inflammatory cytokine gene expression was not significantly changed by either 2 hrs of the SDR stressor or 2 hrs of the stressor repeated on 6 consecutive days. Thus, the results of this study indicate that it is not likely that stressor-induced colonic inflammation is responsible for altering the abundances of the indigenous colonic mucosa-associated microbiota.

The effects of SDR on male mouse immunological, physiological, and behavioral functioning have been well characterized over the past fifteen years [47-49]. The consistent activation of the HPA axis and the SNS makes SDR an ideal method of inducing an acute stress response in male mice. While it would be desirable to determine the effects on female mice as well, aggression in female mice is too low to induce a physiological stress response in this paradigm. Other stressor paradigms, however, have shown that stressor exposure in a lab setting can also affect the microbiota composition of female mice [50,51]. Thus, it is likely that a social stressor in female mice would also impact the lactobacilli, as well as *Porphyromonadaceae* and *Parabacteroides*. Interestingly, studies have shown that male and female microbiota can evoke different levels of host sex hormone release and immunological outputs that can then feedback and alter microbial composition [52]. Future studies comparing the microbiota, mucosal immunity and endocrine responses in both male and female mice are likely to demonstrate additional multidirectional interactions between host physiology and the microbiota. This requires further study.

## Conclusions

Gut microbes have important effects on the host especially when they are associated with the mucosa. Previous studies have found that stressor exposure can affect the microbiota, in particular the lactobacilli. However, many of these studies focused on microbes that are shed in the stool during prolonged or chronic stressors [19,23,31,50]. It was not previously clear whether the effects of stress on the microbiota were limited to changes in the number of bacteria shed in the stool, nor whether short lasting acute stressors could also impact the microbiota. The present study demonstrates that exposure to as little as 2 hrs of a social stressor is sufficient to significantly affect some populations of the colonic mucosa-associated microbiota. This builds upon previous work by showing that exposure to the social stressor affects multiple regions (e.g. colon, cecum) and niches (e.g. lumen, mucosa-associated) of the GI tract, as well as two different strains of mice, suggesting that the effect of social stresss exposure upon the microbiota is a universal process, as opposed to either an artifact or isolated finding. The mechanisms by which this occurs are not yet understood, but are not likely to involve stressor-induced inflammation in the colon. Future studies should assess the impact of stressor-associated hormones and gut functioning since others have demonstrated that activation of the stress-associated hormone corticotrophin releasing hormone (CRH) alters gut microbiota, an effect that was associated with alterations in intestinal motility [53]. Because the gut microbiota has been associated with diverse diseases that are known to be exacerbated by stressful situations, such as the inflammatory bowel diseases, irritable bowel syndrome, and even multiple sclerosis, it is becoming increasingly important to understand the impact that stress can have on the microbiota and whether stressor-induced alterations in the microbiota are involved in stressor-induced disease exacerbation [54-57].

## Methods

### Mice

Male CD-1 mice and male C57BL/6, aged 6–8 weeks, were ordered from Charles River Laboratories (Wilmington, MA; Raleigh, NC). The mice were housed in groups of 2 or 3 mice per cage in an approved vivarium and were allowed to habituate to the vivarium for one week prior to testing. The cages were kept in an approved vivarium with a 12:12 hour light–dark schedule with lights on 0600 to 1800. Food and water was available *ad libitum*. The Ohio State University’s Animal Care and Use Committee approved all experimental procedures (Protocol 2009A0235-R1). CD-1 mice were used in cytokine and bacterial qPCR experiments. C57BL/6 mice were only used in the 454 pyrosequencing analysis and the cytokine and bacterial qPCR experiments.

### Social disruption stressor

The SDR experiments were performed as described previously [58]. Briefly, an aggressive male CD-1 retired breeder, termed the aggressor, was placed into a cage with younger C57BL/6 resident mice at 1700 hours, which represents the beginning of the mouse active cycle. The aggressor repeatedly attacked and defeated the C57BL/6 test mice over the course of the 2 hour SDR cycle. If the aggressor did not begin to attack the test mice within 10 min of placement in the cage, it was removed and another aggressor was put in. Wounding was monitored throughout the SDR cycle. Only slight superficial wounds were allowed. If wounds that penetrated the cutaneous layer developed over the course of SDR, those test mice were removed from the study. While it would be desirable to test the effects of the stressor on both male and female mice, female mice do not fight in this paradigm. Thus, all analyses were limited to male mice.

### Tissue removal

After exposure to the SDR stressor (a single 2 hr cycle for pyrosequencing, a single 2 hr cycle or 2 hr cycles repeated on 6 consecutive days for qPCR), or at the equivalent time in non-stressed HCC mice, mice were euthanized using CO_2_ asphyxiation. Colons were removed aseptically. Luminal contents were removed by cutting into the tissue where liquid and/or solid contents rested, and gently extracting with forceps. This represented the non-mucosa associated populations. Scraping/tissue squeezing was not performed so as not to disturb the mucosa-associated populations, which are adhered to an easily disturbed mucous layer. The colonic tissue and remaining adherent microbiota populations were placed into a microcentrifuge tube and snap frozen in liquid nitrogen. All samples were stored at -80°C until processing. Studies involving 454 pyrotag sequencing utilized the C57BL/6 colonic tissue that was collected after a single 2 hr cycle of SDR, with n = 5 per group (HCC vs. SDR). Animals were kept 3 and 2 mice/cage to minimize cage effects. Studies involving bacterial quantification PCR utilized colonic tissue collected at 0 days of SDR (n = 16 C57BL6, n = 21 CD-1), following a single 2 hr cycle of SDR (n = 7 C57BL/6, n = 15 CD-1) as well as samples collected after SDR repeated on 6 consecutive days (n = 8 C57BL/6, n = 6 CD-1). All experiments were n = 3 or 4 per group. The single 2 hour cycle of SDR in C57BL/6 was replicated once, and six repeated exposures of SDR on C57BL/6 was replicated twice. The single 2 hour cycle of SDR in CD1 mice was replicated four times, and six repeated exposures of SDR on CD1 mice was replicated once. Successive replicates were performed to reduce high variability between samples in bacterial qPCR. No-SDR-exposure groups were sacrificed alongside both single 2 hr cycle of SDR and 6 repeated cycles of SDR groups and combined during statistical analyses, resulting in an increased number of replications over test groups.

### bTEFAP pyrosequencing

Amplicon pyrosequencing (bTEFAP®) was originally described by Dowd et al. on C57BL/6 mice colonic samples and has been utilized in describing a wide range of environmental and health related microbiomes including the intestinal populations of a variety of sample types and environments [59-61]. In this protocol, a 1-mm segment was used from the center of the entire colonic length. In a modified version of this process, 16S universal Eubacterial primers 530 F 5’GTGCCAGCMGCNGCGG and 1100R 5’GGGTTNCGNTCGTTR were used in a single-step 30 cycle PCR using HotStarTaq Plus Master Mix Kit (Qiagen, Valencia, CA). The following conditions were used: 94°C for 3 minutes, followed by 28 cycles of 94°C for 30 seconds; 53°C for 40 seconds and 72°C for 1 minute; after which a final elongation step at 72°C for 5 minutes was performed. Following PCR, all amplicon products from different samples were mixed in equal concentrations and purified using Agencourt Ampure beads (Agencourt Bioscience Corporation, MA, USA). Samples were sequenced utilizing Roche 454 FLX titanium instruments and reagents and following manufacturer’s guidelines.

### Microbial community analysis

The returned sequences were processed and analyzed using Quantitative Insights Into Microbial Ecology (QIIME) (version 1.4.0) [62] according to standard protocols. Filtering passed sequences based on: length between 200 and 1000 bp, zero ambiguous bases or primer mismatches, and no homopolymer runs greater than 6. 97.9% of the sequences passed quality filtering for further analysis. After filtering, there was an average of 10496 sequences per sample. Sequences with 97% similarity were grouped into OTU using UCLUST [63]. Representative sequences from each OTU were aligned against the Greengenes core reference alignment using PyNAST [64,65]. The RDP Classifier was used to assign taxonomy to each representative sequence against the RDP database using standard options [66]. The minimum confidence threshold for taxonomic assignment was 0.80. OTUs were considered unclassified if there was not a strong match within this confidence interval for the representative sequence within the RDP database. A phylogenetic tree was built using FastTree from the aligned OTU representative sequences for determining UniFrac distances between samples [67].

### Quantification of bacterial groups using quantitative polymerase chain reaction (qPCR)

#### DNA extraction

50 mg of tissue, taken from the ascending colon portion of the colonic tract, were homogenized in liquid nitrogen in a mortar and pestle and transferred to a microcentrifuge tube. The samples were incubated for 75 min in lysozyme buffer (20 mg/mL lysozyme, 20 mM Tris–HCl, 2 mM EDTA, 1.2% Triton) at 37°C to weaken the cell wall of Gram-positive bacteria. Following this, DNA was isolated using a modified version of the Qiagen DNA Mini Isolation Kit protocol (Qiagen, Germany). In short, 100 μL of Buffer ATL and 20 μL of proteinase K were added to the microcentrifuge tube and incubated for 3.5 hr at 56°C, with manual vortexing at 30 minute intervals for the duration of the incubation. The samples were heated at 85°C for 10 min to inactivate proteinase K and then transferred into a new tube containing 20 μL of proteinase K and 200 μL of Buffer AL. The samples were incubated at 56°C for 30 min followed by 95°C for 10 min. 200 μL of absolute ethanol was then added to the sample and the sample was added to a QIAamp spin column. After sample washing, the DNA from each sample was recovered into 100 μL of Buffer AE using a QIAamp spin column per manufacturer’s protocols. The DNA extracts were stored at -80°C until analysis was performed. Genomic DNA from pure bacterial cultures was isolated essentially as described above, but using a Qiagen protocol for the isolation of genomic DNA from Gram-positive bacteria and shorter (45 minutes instead of 75 minutes) incubation in the lysozyme buffer.

#### Sample derived standards

The method of Chen et al. [68] was adapted for the generation of sample-derived qPCR standards [68]. Briefly, equivalent mass amounts of colonic DNA from each sample were pooled as the template for PCR to obtain even bacterial representation in order to generate standards for bacterial copy number quantification. DNA was quantified using the Quant-it PicoGreen kit (Life Technologies, Grand Island, New York). Amplification PCR targeting specific genera or species-level 16S rRNA was performed to obtain standard amplicons based upon the representative populations within each sample. PCR was achieved using an ABIPrism 7000 thermocycler. The primer sets are shown in Table 5. The standard amplification PCR thermoprofile for the genus *Lactobacillus* consisted of initial denaturation at 94°C for 5 min, followed by 35 cycles of 94°C for 30 sec, 58°C for 30 sec, and 72°C for 40 sec, and final extension at 72°C for 10 min. For *L. reuteri* PCR, the thermoprofile used was the same as those for the genus-specific except for a higher primer annealing temperature (60°C instead of 58°C). The *Bacteroides-Prevotella-Porphyromonas* standard amplification thermoprofile was 1 cycle at 95°C for 5 min, followed by 35 cycles of 95°C for 20 s, 68°C for 30 s, and 72° for 55 s, with 1 final annealing cycle of 72°C for 5 min. The *Parabacteroides distasonis* standard amplification thermoprofile was 1 cycle at 94°C for 5 min, followed by 30 cycles of 94°C for 30 s, 60°C for 30 s, and 72°C for 40 s, with 1 final annealing cycle at 72°C for 8 min. After the amplification PCR, the PCR product was purified using the Qiagen PCR Purification Kit and quantified by Quant-it PicoGreen kit to determine copy number of the community standards.

**Table 5:** PCR primers and probes

*Lactobacillus genus*	Forward	AGCAGTAGGGAATCTTCCA
	Reverse	CACCGCTACACATGGAG
*Lactobacillus reuteri*	Forward	CAGACAATCTTTGATTGTTTAG
	Reverse	GCTTGTTGGTTTGGGCTCTTC
*Parabacteroides distasonis*	Forward	TGCCTATCAGAGGGGGATAAC
	Reverse	GCAAATATTCCCATGCGGGAT
*Porphyromonas-Bacteroides-Prevotella*	Forward	GGTGTCGGCTTAAGTGCCAT
	Reverse	CGGA(C/T)GTAAGGGCCGTGC
TNF-α	Forward	CTGTCTACTGAACTTCGGGGTGAT
	Reverse	GCTCTGGGCCATAGAACTGATG
	Probe	ATGAGAAGTTCCCAAATGGCCTCCCTC
IL-1β	Forward	GGCCTCAAAGGAAAGAATCTATACC
	Reverse	GTATTGCTTGGGATCCACACTCT
	Probe	ATGAAAGACGGCACACCCACCCTG
iNOS	Forward	CAGCTGGGCTGTACAAACCTT
	Reverse	TGAATGTGATGTTTGCTTCGG
	Probe	CGGGCAGCCTGTGAGACCTTTGA

A standard curve for absolute quantification was created from the previously amplified standards to encompass 10^1^-10^8^ copies per reaction. The population of total lactobacilli and *L. reuteri* was separately quantified using respective specific primers and SYBR Green against these dilution series as described previously [69]. qPCR thermoprofiles for *Lactobacillus* and *L. reuteri* were 1 cycle at 94°C for 5 min, followed by 40 cycles of 94°C for 30 s, specific annealing temperature for 30 s, and 72°C for 40 s. Annealing temperature for *Lactobacillus* was 58°C and *L. reuteri* was 60°C. qPCR thermoprofile for *P. distasonis* was 1 cycle at 50°C for 2 min, 1 cycle at 95°C for 10 min, then 40 cycles at 95°C for 15 s and 60°C for 60 s. qPCR thermoprofile for *Bacteroides-Prevotella-Porphyromonas* was 1 cycle at 95°C for 5 min, followed by 40 cycles at 95°C for 15 s, 68°C for 20 s, and 72°C for 30 s. Abundance of bacterial groups (copies of 16 s rRNA gene per gram/sample) was computed based on the copies of qPCR reaction and the number of reactions that could be performed with the DNA derived from 1 g of each tissue sample. The detection limit were as follows: total lactobacilli and *Bacteroides-Prevotella-Porphyromonas* was ~4.0 log copies/gram of wet tissue (log_10_), *L. reuteri* was ~4.5 log copies/gram of wet tissue (log_10_), *P. distasonis* was ~5.0 log copies/gram of wet tissue. Primer sequences have been previously published and are listed in Table 5[70-74].

### Quantification of colonic cytokine and inflammatory mediator mRNA using quantitative RT-PCR

Total RNA was isolated from whole colonic tissue samples (~120 mg) using TRI-zol (Invitrogen, Carlsbad, California) according to manufacturer’s instructions. The extracted RNA was quantified via spectrophotometry, and converted to cDNA using the Reverse Transcription System (Promega, Madison, WI). qPCR was then completed using a master mix containing 2× Universal TaqMan master mix (Life Technologies, Grand Island, New York), 0.9 μM (each) forward and reverse primers (see Table 5), and 0.250 μg sample cDNA. 18S was used as the housekeeping gene. The PCR was performed using a Prism 7000 sequence detection system with the following thermoprofile: 2 min at 50°C, 10 min at 95°C, and then 40 amplification cycles of 15 s at 95°C and 1 min at 60°C. The relative amount of mRNA was determined using the comparative cycle threshold (*C*_*T*_) method as previously described [75]. Non-stressed HCC control samples were used as baseline controls and were set at a value of 1. All other samples are based on a fold change from these control samples. Primer sequences have been previously published and are listed in Table 5[76]. All groups were n = 3 – 5, using a single experiment without replicates.

### Statistical analyses

Alpha diversity measurements, including Shannon diversity index, equitability (evenness) and Chao (richness) were computed using QIIME on the groups (HCC control vs. SDR Stressor) [77]. Non-parametric T-tests with 999 Monte Carlo permutations were used on these alpha diversity measurements to test for significance at a sampling depth of 4805 sequences per sample.

Taxonomic abundances attained from QIIME were compared between the two groups of mice using non-parametric Mann–Whitney U Tests. PCoA of unweighted UniFrac distance matrices was used to determine clustering between the two groups (HCC control vs. SDR Stressor) [78]. Analysis of Similarity (ANOSIM), a beta-diversity statistic that is available through the vegan package of R and accessible with QIIME, was used to calculate statistical significance between the distance matrices of groups at 999 permutations [79,80]. All of these analyses were performed in QIIME.

Differences in bacterial abundances were determined using non-parametric Kruskal Wallis tests with the cycle of SDR (i.e., 0, 1, or 6 cycles) as the single factor. Mann–Whitney U tests were performed *a priori.* Changes in cytokine gene expression were determined using a two-factor ANOVA with group (HCC control vs. SDR Stressor) and Day (1 Day vs. 6 Days) as the two independent factors. Protected means comparisons were used as post-hoc tests. In all cases, α was set at 0.05, while tendency was declared at 0.05 < p < 0.10. These tests were performed using SPSS v.21 (IBM, Chicago, IL).

### Availability of supporting data

The sequences supporting the results of this article are available in the NCBI Sequence Read Archive under the study accession number SRP035598 (http://www.ncbi.nlm.nih.gov/sra/?term=SRP035598).

## Abbreviations

GI: Gastrointestinal; SDR: Social disruption; qPCR: Quantitative real-time polymerase chain reaction; HPA: Hypothalamic-pituitary-adrenal axis; OTU: Operational taxonomic unit; UPGMA: Unweighted pair-group method with arithmetic mean; ANOSIM: Analysis of similarity; HCC: Home cage control; IL-1β: Interleukin 1-beta; TNF-α: Tumor necrosis factor-alpha; iNOS: Inducible nitric oxide synthase; DSS: Dextran sodium sulfate; CRH: Corticotrophin-releasing hormone; bTEFAP: Bacterial tag-encoded FLX amplicon pyrosequencing; QIIME: Quantitative Insights Into Microbial Ecology; PCoA: Principle coordinates analysis.

## Competing interests

The authors declare they have no competing interests.

## Authors’ contributions

JDG performed all mouse experiments, analyzed 454 sequencing data, and drafted the manuscript. MCN assisted on the 454 sequencing data. ZY assisted with the qPCR method and PCR data analysis. SED performed 454 pyrosequencing. JW assisted with the qPCR method and data analysis. PSK assisted the qPCR method and PCR data analysis. ML designed the mouse experiments. MTB designed the mouse experiments, analyzed 454 sequencing data, and drafted the manuscript. All others edited and approved the final manuscript.

## References

[B1] Turnbaugh PJ Ley RE Hamady M Fraser-Liggett CM Knight R Gordon JI The human microbiome project Nature 2007 449 7164 804 810 17943116 10.1038/nature06244 PMC3709439

[B2] Lozupone CA Stombaugh J Gonzalez A Ackermann G Wendel D Vazquez-Baeza Y Jansson JK Gordon JI Knight R Meta-analyses of studies of the human microbiota Genome Res 2013 23 10 1704 1714 23861384 10.1101/gr.151803.112 PMC3787266

[B3] Reinhardt C Bergentall M Greiner TU Schaffner F Ostergren-Lunden G Petersen LC Ruf W Backhed F Tissue factor and PAR1 promote microbiota-induced intestinal vascular remodelling Nature 2012 483 7391 627 631 22407318 10.1038/nature10893 PMC3885420

[B4] Backhed F Ding H Wang T Hooper LV Koh GY Nagy A Semenkovich CF Gordon JI The gut microbiota as an environmental factor that regulates fat storage Proc Natl Acad Sci U S A 2004 101 44 15718 15723 15505215 10.1073/pnas.0407076101 PMC524219

[B5] Hooper LV Wong MH Thelin A Hansson L Falk PG Gordon JI Molecular analysis of commensal host-microbial relationships in the intestine Science 2001 291 5505 881 884 11157169 10.1126/science.291.5505.881

[B6] Atarashi K Tanoue T Shima T Imaoka A Kuwahara T Momose Y Cheng G Yamasaki S Saito T Ohba Y Taniguchi T Takeda K Hori S Ivanov II Umesaki Y Itoh K Honda K Induction of colonic regulatory T cells by indigenous Clostridium species Science 2011 331 6015 337 341 21205640 10.1126/science.1198469 PMC3969237

[B7] El Aidy S van Baarlen P Derrien M Lindenbergh-Kortleve DJ Hooiveld G Levenez F Dore J Dekker J Samsom JN Nieuwenhuis EE Kleerebezem M Temporal and spatial interplay of microbiota and intestinal mucosa drive establishment of immune homeostasis in conventionalized mice Mucosal Immunol 2012 5 5 567 579 22617837 10.1038/mi.2012.32

[B8] Williams AM Probert CS Stepankova R Tlaskalova-Hogenova H Phillips A Bland PW Effects of microflora on the neonatal development of gut mucosal T cells and myeloid cells in the mouse Immunology 2006 119 4 470 478 16995882 10.1111/j.1365-2567.2006.02458.x PMC2265821

[B9] Rehman A Sina C Gavrilova O Hasler R Ott S Baines JF Schreiber S Rosenstiel P Nod2 is essential for temporal development of intestinal microbial communities Gut 2011 60 10 1354 1362 21421666 10.1136/gut.2010.216259

[B10] Couturier-Maillard A Secher T Rehman A Normand S De Arcangelis A Haesler R Huot L Grandjean T Bressenot A Delanoye-Crespin A Gaillot O Schreiber S Lemoine Y Ryffel B Hot D Nùñez G Chen G Rosenstiel P Chamaillard M NOD2-mediated dysbiosis predisposes mice to transmissible colitis and colorectal cancer J Clin Invest 2013 123 2 700 711 23281400 10.1172/JCI62236 PMC3561825

[B11] Xu J Gordon JI Honor thy symbionts Proc Natl Acad Sci U S A 2003 100 18 10452 10459 12923294 10.1073/pnas.1734063100 PMC193582

[B12] Eckburg PB Bik EM Bernstein CN Purdom E Dethlefsen L Sargent M Gill SR Nelson KE Relman DA Diversity of the human intestinal microbial flora Science 2005 308 5728 1635 1638 15831718 10.1126/science.1110591 PMC1395357

[B13] Ley RE Backhed F Turnbaugh P Lozupone CA Knight RD Gordon JI Obesity alters gut microbial ecology Proc Natl Acad Sci U S A 2005 102 31 11070 11075 16033867 10.1073/pnas.0504978102 PMC1176910

[B14] Nava GM Friedrichsen HJ Stappenbeck TS Spatial organization of intestinal microbiota in the mouse ascending colon ISME J 2011 5 4 627 638 20981114 10.1038/ismej.2010.161 PMC3105732

[B15] Bailey MT Dowd SE Galley JD Hufnagle AR Allen RG Lyte M Exposure to a social stressor alters the structure of the intestinal microbiota: implications for stressor-induced immunomodulation Brain Behav Immun 2011 25 3 397 407 21040780 10.1016/j.bbi.2010.10.023 PMC3039072

[B16] Turnbaugh PJ Ley RE Mahowald MA Magrini V Mardis ER Gordon JI An obesity-associated gut microbiome with increased capacity for energy harvest Nature 2006 444 7122 1027 1031 17183312 10.1038/nature05414

[B17] Chang JY Antonopoulos DA Kalra A Tonelli A Khalife WT Schmidt TM Young VB Decreased diversity of the fecal Microbiome in recurrent Clostridium difficile-associated diarrhea J Infect Dis 2008 197 3 435 438 18199029 10.1086/525047

[B18] Sekirov I Tam NM Jogova M Robertson ML Li Y Lupp C Finlay BB Antibiotic-induced perturbations of the intestinal microbiota alter host susceptibility to enteric infection Infect Immun 2008 76 10 4726 4736 18678663 10.1128/IAI.00319-08 PMC2546810

[B19] Bailey MT Coe CL Maternal separation disrupts the integrity of the intestinal microflora in infant rhesus monkeys Dev Psychobiol 1999 35 2 146 155 10461128

[B20] Mackos AR Eubank TD Parry NM Bailey MT Probiotic Lactobacillus reuteri attenuates the stressor-enhanced severity of Citrobacter rodentium infection Infect Immun 2013 81 9 3253 3263 23798531 10.1128/IAI.00278-13 PMC3754198

[B21] Schreiber O Petersson J Phillipson M Perry M Roos S Holm L Lactobacillus reuteri prevents colitis by reducing P-selectin-associated leukocyte- and platelet-endothelial cell interactions Am J Physiol Gastrointest Liver Physiol 2009 296 3 G534 G542 19147805 10.1152/ajpgi.90470.2008

[B22] Barouei J Moussavi M Hodgson DM Effect of maternal probiotic intervention on HPA axis, immunity and gut microbiota in a rat model of irritable bowel syndrome PLoS One 2012 7 10 e46051 23071537 10.1371/journal.pone.0046051 PMC3469551

[B23] Bailey MT Dowd SE Parry NM Galley JD Schauer DB Lyte M Stressor exposure disrupts commensal microbial populations in the intestines and leads to increased colonization by Citrobacter rodentium Infect Immun 2010 78 4 1509 1519 20145094 10.1128/IAI.00862-09 PMC2849416

[B24] Hanke ML Powell ND Stiner LM Bailey MT Sheridan JF Beta adrenergic blockade decreases the immunomodulatory effects of social disruption stress Brain Behav Immun 2012 26 7 1150 1159 22841997 10.1016/j.bbi.2012.07.011 PMC3506115

[B25] Bernstein CN Singh S Graff LA Walker JR Miller N Cheang M A prospective population-based study of triggers of symptomatic flares in IBD Am J Gastroenterol 2010 105 9 1994 2002 20372115 10.1038/ajg.2010.140

[B26] Dickhaus B Mayer EA Firooz N Stains J Conde F Olivas TI Fass R Chang L Mayer M Naliboff BD Irritable bowel syndrome patients show enhanced modulation of visceral perception by auditory stress Am J Gastroenterol 2003 98 1 135 143 12526949 10.1111/j.1572-0241.2003.07156.x

[B27] Lee KJ Tack J Altered intestinal microbiota in irritable bowel syndrome Neurogastroenterol Motil 2010 22 5 493 498 20414959 10.1111/j.1365-2982.2010.01482.x

[B28] Sekirov I Russell SL Antunes LC Finlay BB Gut microbiota in health and disease Physiol Rev 2010 90 3 859 904 20664075 10.1152/physrev.00045.2009

[B29] Sartor RB Microbial influences in inflammatory bowel diseases Gastroenterology 2008 134 2 577 594 18242222 10.1053/j.gastro.2007.11.059

[B30] Garrett WS Lord GM Punit S Lugo-Villarino G Mazmanian SK Ito S Glickman JN Glimcher LH Communicable ulcerative colitis induced by T-bet deficiency in the innate immune system Cell 2007 131 1 33 45 17923086 10.1016/j.cell.2007.08.017 PMC2169385

[B31] Bailey MT Lubach GR Coe CL Prenatal stress alters bacterial colonization of the gut in infant monkeys J Pediatr Gastroenterol Nutr 2004 38 4 414 421 15085020 10.1097/00005176-200404000-00009

[B32] Carroll IM Chang YH Park J Sartor RB Ringel Y Luminal and mucosal-associated intestinal microbiota in patients with diarrhea-predominant irritable bowel syndrome Gut pathogens 2010 2 1 19 21143915 10.1186/1757-4749-2-19 PMC3018384

[B33] Bajaj JS Hylemon PB Ridlon JM Heuman DM Daita K White MB Monteith P Noble NA Sikaroodi M Gillevet PM Colonic mucosal microbiome differs from stool microbiome in cirrhosis and hepatic encephalopathy and is linked to cognition and inflammation Am J Physiol Gastrointest Liver Physiol 2012 303 6 G675 G685 22821944 10.1152/ajpgi.00152.2012 PMC3468538

[B34] Van den Abbeele P Van de Wiele T Verstraete W Possemiers S The host selects mucosal and luminal associations of coevolved gut microorganisms: a novel concept FEMS Microbiol Rev 2011 35 4 681 704 21361997 10.1111/j.1574-6976.2011.00270.x

[B35] Knowles SR Nelson EA Palombo EA Investigating the role of perceived stress on bacterial flora activity and salivary cortisol secretion: a possible mechanism underlying susceptibility to illness Biol Psychol 2008 77 2 132 137 18023961 10.1016/j.biopsycho.2007.09.010

[B36] Tannock GW Savage DC Influences of dietary and environmental stress on microbial populations in the murine gastrointestinal tract Infect Immun 1974 9 3 591 598 4593471 10.1128/iai.9.3.591-598.1974 PMC414848

[B37] Friswell MK Gika H Stratford IJ Theodoridis G Telfer B Wilson ID McBain AJ Site and strain-specific variation in gut microbiota profiles and metabolism in experimental mice PLoS One 2010 5 1 e8584 20052418 10.1371/journal.pone.0008584 PMC2798964

[B38] Hildebrand F Nguyen TL Brinkman B Yunta RG Cauwe B Vandenabeele P Liston A Raes J Inflammation-associated enterotypes, host genotype, cage and inter-individual effects drive gut microbiota variation in common laboratory mice Genome Biol 2013 14 1 R4 23347395 10.1186/gb-2013-14-1-r4 PMC4053703

[B39] Bouwknecht JA Paylor R Behavioral and physiological mouse assays for anxiety: a survey in nine mouse strains Behav Brain Res 2002 136 2 489 501 12429412 10.1016/s0166-4328(02)00200-0

[B40] Julio-Pieper M O'Mahony CM Clarke G Bravo JA Dinan TG Cryan JF Chronic stress-induced alterations in mouse colonic 5-HT and defecation responses are strain dependent Stress 2012 15 2 218 226 21875301 10.3109/10253890.2011.607524

[B41] Jang SE Hyam SR Han MJ Kim SY Lee BG Kim DH Lactobacillus brevis G-101 ameliorates colitis in mice by inhibiting NF-kappaB, MAPK and AKT pathways and by polarizing M1 macrophages to M2-like macrophages J Appl Microbiol 2013 115 3 888 896 23742179 10.1111/jam.12273

[B42] Cuevas M Flores I Thompson KJ Ramos-Ortolaza DL Torres-Reveron A Appleyard CB Stress exacerbates endometriosis manifestations and inflammatory parameters in an animal model Reprod Sci 2012 19 8 851 862 22527982 10.1177/1933719112438443 PMC4046310

[B43] Hoffmann C Hill DA Minkah N Kirn T Troy A Artis D Bushman F Community-wide response of the gut microbiota to enteropathogenic Citrobacter rodentium infection revealed by deep sequencing Infect Immun 2009 77 10 4668 4678 19635824 10.1128/IAI.00493-09 PMC2747949

[B44] Craven M Egan CE Dowd SE McDonough SP Dogan B Denkers EY Bowman D Scherl EJ Simpson KW Inflammation drives dysbiosis and bacterial invasion in murine models of ileal Crohn's disease PLoS One 2012 7 7 e41594 22848538 10.1371/journal.pone.0041594 PMC3404971

[B45] Lupp C Robertson ML Wickham ME Sekirov I Champion OL Gaynor EC Finlay BB Host-mediated inflammation disrupts the intestinal microbiota and promotes the overgrowth of Enterobacteriaceae Cell Host Microbe 2007 2 2 119 129 18005726 10.1016/j.chom.2007.06.010

[B46] Collins SM McHugh K Jacobson K Khan I Riddell R Murase K Weingarten HP Previous inflammation alters the response of the rat colon to stress Gastroenterology 1996 111 6 1509 1515 8942729 10.1016/s0016-5085(96)70012-4

[B47] Quan N Avitsur R Stark JL He L Lai W Dhabhar F Sheridan JF Molecular mechanisms of glucocorticoid resistance in splenocytes of socially stressed male mice J Neuroimmunol 2003 137 1–2 51 58 12667647 10.1016/s0165-5728(03)00042-0

[B48] Kinsey SG Bailey MT Sheridan JF Padgett DA Avitsur R Repeated social defeat causes increased anxiety-like behavior and alters splenocyte function in C57BL/6 and CD-1 mice Brain Behav Immun 2007 21 4 458 466 17178210 10.1016/j.bbi.2006.11.001 PMC1941837

[B49] Mays JW Bailey MT Hunzeker JT Powell ND Papenfuss T Karlsson EA Padgett DA Sheridan JF Influenza virus-specific immunological memory is enhanced by repeated social defeat J Immunol 2010 184 4 2014 2025 20083672 10.4049/jimmunol.0900183 PMC3066050

[B50] Bangsgaard Bendtsen KM Krych L Sorensen DB Pang W Nielsen DS Josefsen K Hansen LH Sorensen SJ Hansen AK Gut microbiota composition is correlated to grid floor induced stress and behavior in the BALB/c mouse PLoS One 2012 7 10 e46231 23056268 10.1371/journal.pone.0046231 PMC3462757

[B51] Sun Y Zhang M Chen CC Gillilland M Jr Sun X El-Zaatari M Huffnagle GB Young VB Zhang J Hong SC Chang YM Gumucio DL Owyang C Kao JY Stress-induced corticotropin-releasing hormone-mediated NLRP6 inflammasome inhibition and transmissible enteritis in mice Gastroenterology 2013 144 7 1478 1487 1487 e1471-1478 23470617 10.1053/j.gastro.2013.02.038 PMC3777426

[B52] Markle JG Frank DN Mortin-Toth S Robertson CE Feazel LM Rolle-Kampczyk U von Bergen M McCoy KD Macpherson AJ Danska JS Sex differences in the gut microbiome drive hormone-dependent regulation of autoimmunity Science 2013 339 6123 1084 1088 23328391 10.1126/science.1233521

[B53] Park AJ Collins J Blennerhassett PA Ghia JE Verdu EF Bercik P Collins SM Altered colonic function and microbiota profile in a mouse model of chronic depression Neurogastroenterol Motil 2013 25 9 733 e575 23773726 10.1111/nmo.12153 PMC3912902

[B54] Greene BR Blanchard EB Wan CK Long-term monitoring of psychosocial stress and symptomatology in inflammatory bowel disease Behav Res Ther 1994 32 2 217 226 8155059 10.1016/0005-7967(94)90114-7

[B55] Bennett EJ Tennant CC Piesse C Badcock CA Kellow JE Level of chronic life stress predicts clinical outcome in irritable bowel syndrome Gut 1998 43 2 256 261 10189854 10.1136/gut.43.2.256 PMC1727204

[B56] Berer K Mues M Koutrolos M Rasbi ZA Boziki M Johner C Wekerle H Krishnamoorthy G Commensal microbiota and myelin autoantigen cooperate to trigger autoimmune demyelination Nature 2011 479 7374 538 541 22031325 10.1038/nature10554

[B57] Buljevac D Hop WC Reedeker W Janssens AC van der Meche FG van Doorn PA Hintzen RQ Self reported stressful life events and exacerbations in multiple sclerosis: prospective study BMJ 2003 327 7416 646 14500435 10.1136/bmj.327.7416.646 PMC196389

[B58] Allen RG Lafuse WP Powell ND Webster Marketon JI Stiner-Jones LM Sheridan JF Bailey MT Stressor-induced increase in microbicidal activity of splenic macrophages is dependent upon peroxynitrite production Infect Immun 2012 80 10 3429 3437 22825446 10.1128/IAI.00714-12 PMC3457565

[B59] Dowd SE Callaway TR Wolcott RD Sun Y McKeehan T Hagevoort RG Edrington TS Evaluation of the bacterial diversity in the feces of cattle using 16S rDNA bacterial tag-encoded FLX amplicon pyrosequencing (bTEFAP) BMC Microbiol 2008 8 125 18652685 10.1186/1471-2180-8-125 PMC2515157

[B60] Callaway TR Dowd SE Edrington TS Anderson RC Krueger N Bauer N Kononoff PJ Nisbet DJ Evaluation of bacterial diversity in the rumen and feces of cattle fed different levels of dried distillers grains plus solubles using bacterial tag-encoded FLX amplicon pyrosequencing J Anim Sci 2010 88 12 3977 3983 20729286 10.2527/jas.2010-2900

[B61] Williams WL Tedeschi LO Kononoff PJ Callaway TR Dowd SE Karges K Gibson ML Evaluation of in vitro gas production and rumen bacterial populations fermenting corn milling (co)products J Dairy Sci 2010 93 10 4735 4743 20855008 10.3168/jds.2009-2920

[B62] Caporaso JG Kuczynski J Stombaugh J Bittinger K Bushman FD Costello EK Fierer N Pena AG Goodrich JK Gordon JI Huttley GA Kelley ST Knights D Koenig JE Ley RE Lozupone CA McDonald D Muegge BD Pirrung M Reeder J Sevinsky JR Turnbaugh PJ Walters WA Widmann J Yatsunenko T Zaneveld J Knight R QIIME allows analysis of high-throughput community sequencing data Nat Methods 2010 7 5 335 336 20383131 10.1038/nmeth.f.303 PMC3156573

[B63] Edgar RC Search and clustering orders of magnitude faster than BLAST Bioinformatics 2010 26 19 2460 2461 20709691 10.1093/bioinformatics/btq461

[B64] DeSantis TZ Hugenholtz P Larsen N Rojas M Brodie EL Keller K Huber T Dalevi D Hu P Andersen GL Greengenes, a chimera-checked 16S rRNA gene database and workbench compatible with ARB Appl Environ Microbiol 2006 72 7 5069 5072 16820507 10.1128/AEM.03006-05 PMC1489311

[B65] Caporaso JG Bittinger K Bushman FD DeSantis TZ Andersen GL Knight R PyNAST: a flexible tool for aligning sequences to a template alignment Bioinformatics 2010 26 2 266 267 19914921 10.1093/bioinformatics/btp636 PMC2804299

[B66] Wang Q Garrity GM Tiedje JM Cole JR Naive Bayesian classifier for rapid assignment of rRNA sequences into the new bacterial taxonomy Appl Environ Microbiol 2007 73 16 5261 5267 17586664 10.1128/AEM.00062-07 PMC1950982

[B67] Price MN Dehal PS Arkin AP FastTree 2–approximately maximum-likelihood trees for large alignments PLoS One 2010 5 3 e9490 20224823 10.1371/journal.pone.0009490 PMC2835736

[B68] Chen J Yu Z Michel FC Jr Wittum T Morrison M Development and application of real-time PCR assays for quantification of erm genes conferring resistance to macrolides-lincosamides-streptogramin B in livestock manure and manure management systems Appl Environ Microbiol 2007 73 14 4407 4416 17496134 10.1128/AEM.02799-06 PMC1932836

[B69] Anderson K Yu Z Chen J Jenkins J Courtney P Morrison M Analyses of Bifidobacterium, Lactobacillus, and total bacterial populations in healthy volunteers consuming calcium gluconate by denaturing gradient gel electrophoresis and real-time PCR Int J Probiotics Prebiotics 2008 3 31 36

[B70] Walter J Hertel C Tannock GW Lis CM Munro K Hammes WP Detection of Lactobacillus, Pediococcus, Leuconostoc, and Weissella species in human feces by using group-specific PCR primers and denaturing gradient gel electrophoresis Appl Environ Microbiol 2001 67 6 2578 2585 11375166 10.1128/AEM.67.6.2578-2585.2001 PMC92910

[B71] Heilig HG Zoetendal EG Vaughan EE Marteau P Akkermans AD de Vos WM Molecular diversity of Lactobacillus spp. and other lactic acid bacteria in the human intestine as determined by specific amplification of 16S ribosomal DNA Appl Environ Microbiol 2002 68 1 114 123 11772617 10.1128/AEM.68.1.114-123.2002 PMC126540

[B72] Rinttila T Kassinen A Malinen E Krogius L Palva A Development of an extensive set of 16S rDNA-targeted primers for quantification of pathogenic and indigenous bacteria in faecal samples by real-time PCR J Appl Microbiol 2004 97 6 1166 1177 15546407 10.1111/j.1365-2672.2004.02409.x

[B73] Brisbin JT Gong J Orouji S Esufali J Mallick AI Parvizi P Shewen PE Sharif S Oral treatment of chickens with lactobacilli influences elicitation of immune responses Clin Vaccine Immunol 2011 18 9 1447 1455 21734067 10.1128/CVI.05100-11 PMC3165221

[B74] Tong J Liu C Summanen P Xu H Finegold SM Application of quantitative real-time PCR for rapid identification of Bacteroides fragilis group and related organisms in human wound samples Anaerobe 2011 17 2 64 68 21439390 10.1016/j.anaerobe.2011.03.004

[B75] Head CC Farrow MJ Sheridan JF Padgett DA Androstenediol reduces the anti-inflammatory effects of restraint stress during wound healing Brain Behav Immun 2006 20 6 590 596 16730942 10.1016/j.bbi.2006.03.007

[B76] Allen RG Lafuse WP Galley JD Ali MM Ahmer BM Bailey MT The intestinal microbiota are necessary for stressor-induced enhancement of splenic macrophage microbicidal activity Brain Behav Immun 2012 26 3 371 382 22100833 10.1016/j.bbi.2011.11.002 PMC3288745

[B77] Shannon CE Weaver W The mathematical theory of communication 1949 Urbana: University of Illinois Press

[B78] Lozupone C Knight R UniFrac: a new phylogenetic method for comparing microbial communities Appl Environ Microbiol 2005 71 12 8228 8235 16332807 10.1128/AEM.71.12.8228-8235.2005 PMC1317376

[B79] Oksanen J Blanchet FG Kindt R Legendre P Minchin PR O’Hara RB Simpson GL Solymos P Henry M Stevens HH Wagner H Vegan: community ecology package 2012 R package version 2.0-3

[B80] Development Core Team R: a language and environment for statistical computing 2011 R Foundation for Statistical Computing, Vienna, Austria. Coventry, United Kingdom R Foundation for Statistical Computing

